# Regression course of ostium granulomas under topical intranasal steroids coverage after endoscopic dacryocystorhinostomy

**DOI:** 10.1038/s41598-024-67620-7

**Published:** 2024-07-24

**Authors:** Shang-Te Ma, Seonae Shin, Apurva Navale, Kyung In Woo

**Affiliations:** 1 Department of Ophthalmology, Taipei Medical University-Shuang Ho Hospital, Ministry of Health and Welfare, New Taipei City, Taiwan; 2 Department of Ophthalmology, Samsung Medical Center, Seoul, Korea; 3 Department of Ophthalmology, Samsung Medical Center, Sungkyunkwan University School of Medicine, 81 Irwon-ro, Gangnam-gu, Seoul, 06351 Korea

**Keywords:** Steroids, Endoscopic dacryocystorhinostomy, DCR, Ostium granulomas, Quality of life, Lacrimal apparatus diseases

## Abstract

This study aimed to elucidate the regression process of ostium granulomas under the usage of intranasal steroid after primary endoscopic dacryocystorhinostomy (DCR). The authors retrospectively reviewed 57 patients (a total of 72 ostia) who had ostium granulomas after primary endoscopic DCR between 2011 and 2015. Topical intranasal steroid spray was applied in all the patients since postoperative day 1. Adjunctive intralesional triamcinolone acetonide injections were administered for extensive and large-sized granulomas that caused impending ostium blockage. Sequential regression of the ostium granulomas and success rates of DCR were assessed using endoscopic photos. The granulomas completely disappeared in 69 (95.8%) ostia, and the average time interval from the surgery to the disappearance was 6.9 ± 2.8 months. Anatomical and functional surgical success rates were 90.3% and 84.7%, respectively. Intralesional steroid injections for ostium granulomas did not alter the outcomes compared to topical intranasal steroid usage significantly (*p* = 0.445). In conclusion, we observed that, by continuing the usage of intranasal steroids, ostium granulomas disappear gradually at postoperative 6 months. The intranasal surgical manipulation of granulomas, which results in more mucosal cicatricial change and impedes patient satisfaction, can be successfully avoided.

## Introduction

Endoscopic dacryocystorhinostomy (DCR) has become a standard procedure for the treatment of nasolacrimal duct obstruction^1,2^, with its failure rate ranging between 10 and 20%^3–8^. Numerous factors related to surgical failures, including younger age, cicatricial ostial closure and common canalicular opening, inadequately sized or placed osteotomy, granulomas formation, turbino-septal and ostio-septal synechiae, and ostium stenosis, were elucidated^8–10^. Among these, ostium granuloma may be one of the evolving anatomical abnormalities and possible modifiable factors after DCR surgery^8–13^. Thus, postoperative inspections of osteotomy status and ostium granuloma periodically are crucial to ensure a proper wound-healing process and surgical success.

Postoperative ostium granulomas were usually managed by surgical excision to prevent further obstruction of the ostium^8,12,14^. Some authors advocated for electrocauterization or power-driven microdebrider to remove the obstructing granuloma tissue based in office settings^15^. In contrast, some authors deferred surgical removal and preferred to adopt intralesional steroid injections to reduce granulation or cicatrix, which was less time-consuming and more tolerable for patients in an office-based setting^11^. However, several injections may be necessary, and frequent follow-up visits also pose patient inconvenience and emotional stress. Although the characteristics, histology patterns, treatment indication, and outcome of ostium granulomas following DCR were well studied and proposed in previous literature^16,17^, to the best of our knowledge, the sequential evolution of non-surgically excised ostium granulomas under topical intranasal steroid usage was not yet provided in details.

In this study, we aimed to demonstrate the sequential evolution process of the non- excised ostium granulomas treated by topical intranasal steroid, and the modifying effects and benefits of topical intranasal steroid usage after endoscopic DCR.

## Methods

We reviewed patients who had ostium granulomas after primary endoscopic DCR between January 2011 and March 2015. All the surgeries were conducted by a single surgeon (K.I.W). Patients who had a short follow-up period (< 6 months), previous sinus surgery, radiation therapy, neoplasm in the lacrimal drainage apparatus, posttraumatic bony deformity, or surgical removal of the granulomas were excluded from further study. Data retrieved from medical records and photo recordings were reviewed retrospectively. The study was approved by the institutional review board of Samsung Seoul Hospital, and it adhered to the tenets of the Declarations of Helsinki. The informed consents have been waived off by the relevant ethical committee owing to the retrospective nature of this current study.

### Surgical methods and postoperative follow-up

All the surgeries were conducted by a 0°, 4 mm endoscope (Karl Storz Endoscopy-America, Inc., El Segundo, CA, U.S.A.) under general anesthesia. The nasal cavity was decongested with an application of neurosurgical cottonoids soaked in 2% lidocaine and 1:50,000 epinephrine before the operation. After localizing the lacrimal sac fossa with a transcanalicular endoilluminator through the upper canaliculus, the nasal mucosa adjacent to the lacrimal sac fossa was infiltrated with local anesthetics containing 1% lidocaine, 0.25% bupivacaine, and 1:100,000 epinephrine. Thus, the nasal mucosa was incised with a sickle knife and partially removed with the Blakesley nasal cutting forceps. The intervening structures between the lacrimal sac and nasal cavity (lacrimal fossa bone, operculum of middle turbinate, uncinate process, or Agger nasi cell) were removed. After adequate exposure of the lacrimal sac, the medial sac wall was incised using a keratome and completely removed. Bicanalicular silicone tubes (Canaliculus Intubation Set®, Beaver-Visitec Inc., Waltham, MA, U.S.A.) were inserted thereafter. At the end of the surgery, the nasal cavity was packed with gentamicin and 40 mg/mL of triamcinolone acetonide (TA) (Tamceton®, Hanall BioPharma, Seoul, Korea) soaked NASOPORE® (Polyganics, Groningen, Netherlands). Fluticasone furoate nasal spray (Avamys®, GlaxoSmithKline plc.) was prescribed once daily for 4 weeks since postoperative day 1. The silicone tubes were left in place and removed at around 3 months postoperatively.

All the patients were examined with 30°, 3 mm nasal endoscope system (Karl Storz Endoscopy-America, Inc., El Segundo, CA, U.S.A.) after administering a lidocaine nasal spray (10% Lidocaine Spray, AstraZeneca, U.K.) postoperatively at 1 week, 1 month, 2 months, 3 months, and 6 months. The ostium and intranasal conditions were documented with photos or videos. If granulomas were detected, we documented the sequential change in appearances on each visit. On examination, the intranasal crust and NASOPORE® remnant were removed with suction devices or nasal Tampon forceps under direct endoscopy. Tear drainage was assessed using the functional endoscopic dye track test after the instillation of 2% topical fluorescein.

### Classifications and management of ostium granulomas

In our study, the ostium granulomas were classified as follows:

1. Location relative to the osteotomy (superior, inferior, anterior, and posterior): we used the ostium as the reference, and divided the periostial area into four quadrants. For granulomas diffusely distributed around the canaliculus intubation set tubes, we defined them as peritubal type. The classification for locations was modified from Ali et al.^14,18^.
2. Shape (pedunculated, sessile, and mixed): pedunculated granulomas were proliferative granulation tissues growing in a mushroom shape, which attached to the nasal mucosa with a single long and thin stalk. In contrast to the pedunculated type, sessile granulomas describe granulation tissues that proliferate in a relatively flattened and broad-based manner. If the proliferative granulation tissue consists of both pedunculated and sessile patterns, it belongs to the mixed type.
3. Number (single, multiple, and diffuse): we sub-categorized the number of granulomas as single or multiple (2–3) types. In contrast, if the granulomas were > 3, which made numbering difficult, we defined them as diffuse type.

We deferred routinely surgical removal of the ostium granulomas and simply adopted monthly observation. Additionally, all the patients were required to keep fluticasone furoate nasal spray once a day (two puffs at a time). For granulomas which grew in size, displaced the inserted canaliculus intubation set tubes, or impending blocking of the ostium, we administered 0.1 mL of intralesional TA injection once as an adjunctive therapy. The patients were then monthly monitored after the TA injection.

### Outcome evaluations and statistical analysis

We recorded the time point when the granulomas developed and sequential changes of the granulomas under persistent usage of topical intranasal steroid usage. The success rates of the DCR were analyzed both anatomically and functionally. The anatomical success of surgical outcome was defined as endoscopic evidence of ostial and canalicular patency on irrigation, while functional success was defined as a free flow of dye into the ostium on functional endoscopic dye test with subjective improvement and resolution of epiphora. Furthermore, we subcategorized the ostium granulomas into TA and non-TA injection groups to evaluate the sequential changes and anatomical and functional success rates between the two groups.

Statistical analysis was performed using SPSS software version 23.0 (SPSS, Inc., Chicago, IL, U.S.A.). Group differences were tested by applying the Pearson’s chi-square or Fisher’s exact test for categorical variables, while independent t-test and analysis of variance (ANOVA) were applied to compare between continuous variables. A clinically significant difference was considered at *p* < 0.05. The data that supported the findings of this study are available on request from the corresponding author (K.I.W.).

## Results

We analyzed 72 ostia with granulomas in 57 patients who were followed up without surgical excision, and with intranasal steroid spray from postoperative day 1. The anatomical and functional success rates in the analyzed 72 ostia were 90.2 and 84.7%, respectively. Demographic data and features of the granulomas are shown in Table 1. The average age was 57.6 ± 14.2 years, with female predominance (n = 48, 84.2%). Fifteen patients had ostium granulomas detected on both sides. The overall follow-up period was 10.9 ± 4.6 months (range: 6–24 months), and the silicone tube removal time point was at 2.9 ± 2.0 months (range: 2–18 months) postoperatively. The most common location was at the superior quadrant of the ostium (n = 28, 38.9%). The pedunculated (n = 35, 48.6%) and sessile-shaped (n = 34, 47.2%) ostium granulomas were observed almost equally, while the mixed type was the least frequent pattern. Most of the ostia had multiple (2–3) identifiable granulomas (n = 33, 45.8%), followed by the single and diffuse pattern. The granuloma location, shape, and number did not affect the final DCR success rate (*p* > 0.05).

**Table 1:** Baseline demographic data and characteristics of 57 patients (72 nasal ostia) with ostium granulomas who received intranasal steroids treatment instead of surgical excision after endoscopic DCR.

Age (years)	57.6 ± 14.2
Sex (female, n, %)	48 (84.2)

Granuloma-related events	Time (months)
Granuloma detection	2.4 ± 0.9 (range: 1–6, median 2.0)
Granuloma disappearance	6.9 ± 2.8 (range: 2–15, median 6.0)

Characteristics of ostium granulomas	Ostia number (%)
All ostium granulomas	72 (100)
Locations	
Superior	28 (38.9)
Anterior	12 (16.7)
Inferior	5 (6.9)
Posterior	2 (2.8)
All quadrants	15 (20.8)
Peritubal	10 (13.9)
Shape	
Pedunculated	35 (48.6)
Sessile	34 (47.2)
Mixed	3 (4.2)
Number	
Single	22 (30.6)
Multiple	33 (45.8)
Diffuse	17 (23.6)

**Figure 1:**
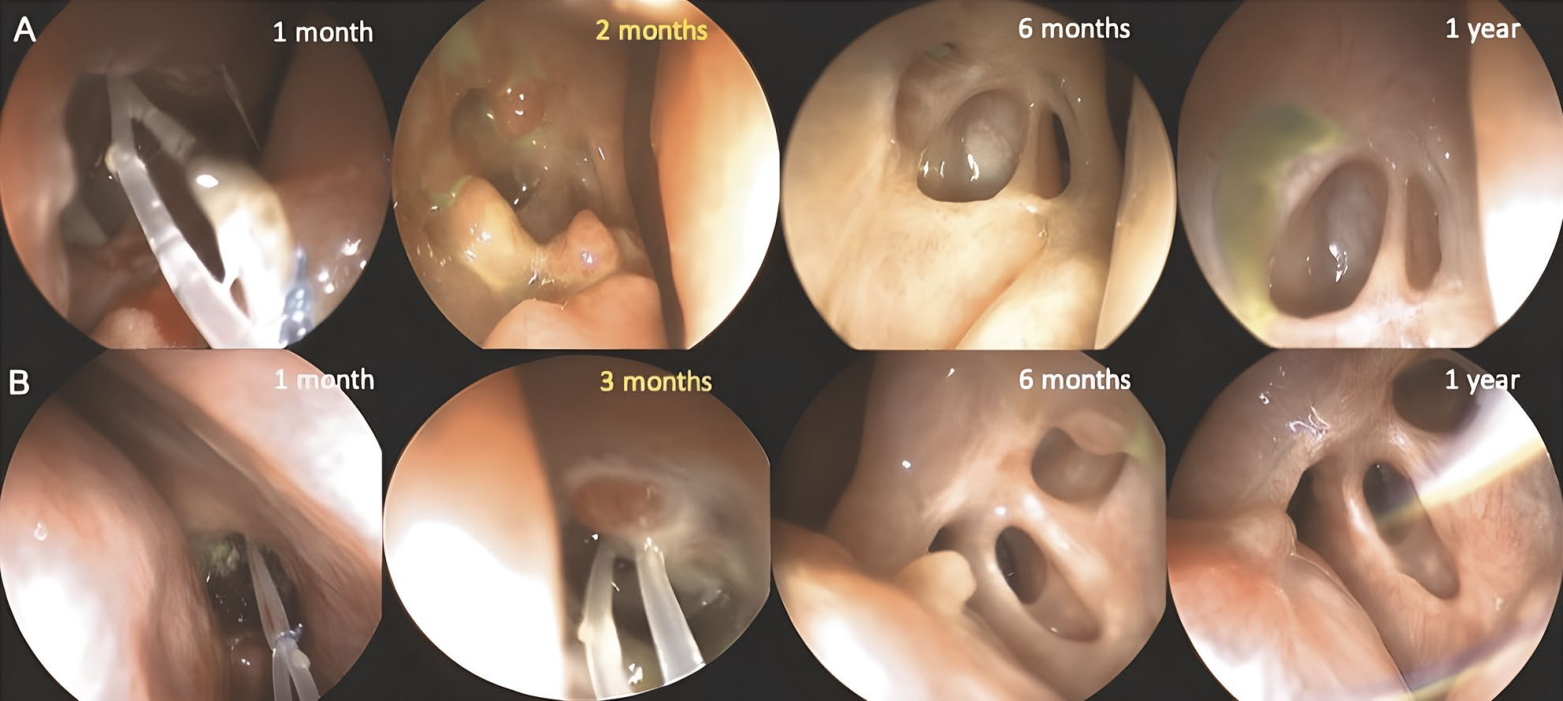
Ostium granulomas spontaneously resolved with topical intranasal steroid therapy. (**A**) Upper panel: A 65-year-old woman received right endoscopic DCR, and she presented with ostium granulomas in all quadrant, mixed pattern, and multiple granulomas at 2 months postoperatively. At 6 months of follow-up, all the ostium granulomas disappeared. (**B**) Lower panel: A 49-year-old woman who underwent left endoscopic DCR showed superior, sessile pattern single granuloma around the ostium at 3 months postoperatively, which showed total resolution at 1 year of follow-up.

**Figure 2:**
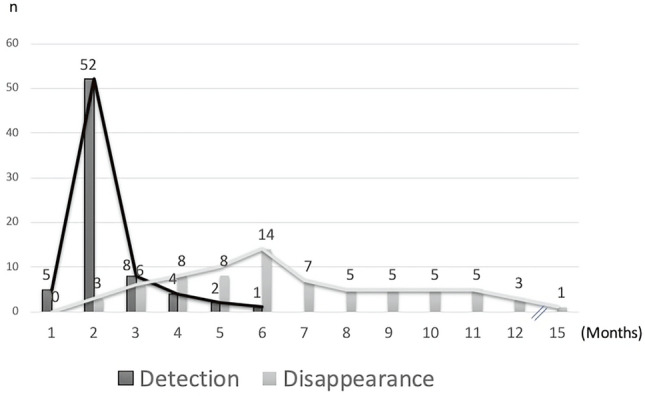
Sequential changes of the ostium granulomas in detection and complete resolution. All the ostia with granulomas (n = 72) developed within 6 months postoperatively (mean: 2.4 ± 0.9 months), with a peak at 2 months postoperatively. The granulomas disappeared in 69 ostia, and more than half of them disappeared within 6 months after the operation (n = 39, 54.2%). The mean time of granuloma disappearance was 6.9 ± 2.8 months (range: 2–15 months).

Regarding detection time, the granulomas were observed within postoperative 2 months in 57 (79.2%) ostia. The mean time interval from operation to granuloma detection was 2.4 ± 0.9 months, and the median time of granuloma detection after operation was 2.0 months (range: 1–6 months). After conservative treatment using intranasal steroid nasal spray with or without intralesional TA injection, the granulomas disappeared within the follow-up period in 69 (95.8%) ostia. One (1.4%) ostium had granulomas that decreased in size until the last follow-up period at 8 months postoperatively. Additionally, two (2.8%) ostia with granulomas remained stationary in size until 6 months postoperatively. However, all three patients were not followed up for any longer period, while all of them had both anatomical and functional success upon the last visits. More than half of the observed ostium granulomas disappeared within 6 months after operation (39 of 69, 56.5%). The mean time of total resolution after DCR surgeries was 6.9 ± 2.8 months (range: 2–15 months) under persistent coverage of intranasal steroid usage. The sequential changes of the ostium granulomas are shown in Figs. 1 and 2. None of the ostium granulomas recurred after resolution.

In our study, we administered additional intralesional TA injections in 13 ostia with topical intranasal steroid spray. The demographics, granuloma characteristics, and surgical success rates were compared between the TA (n = 13) and non-TA (n = 59) injection groups (Table 2). The ostium granulomas that received TA injections were mostly found in the superior quadrant (n = 5, 38.5%), while pedunculated and sessile-shaped granulomas were almost equally distributed for both treatment groups. The multiple (2–3) pattern was more dominant (n = 12, 92.3%, *p* < 0.001) in the TA injection group. The average disappearance time for the granulomas was longer in the TA injection group than in the non-TA group (7.2 and 6.8 months, respectively); however, it did not show statistical significance (*p* = 0.760). Notably, the disappearance rate between the TA and non-TA treatment groups did not show significant dissimilarity (92.3% and 96.6%, respectively, *p* = 0.445). Moreover, the final anatomical and functional surgical success rates did not differ significantly between both groups (*p* = 0.363 and 0.629, respectively). Severe adverse effect related to long-term steroid usage such as iatrogenic Cushing syndrome, severe infection, glaucoma, cataract, or nasal mucosal disorder was not observed in our current study.

**Table 2:** Features of 72 ostia with granulomas subcategorized by TA and non-TA injections groups.

	TA (n = 13)	Non-TA (n = 59)	*p* value
Ostium granulomas received conservative treatments			
Age (years)	56.8 ± 11.0	57.9 ± 13.6	0.555*
Sex (*Male:Female*)	3:10	6:38	0.309^#^
Post-operative events (months)			
Granulomas detection	2.3 ± 0.9	2.4 ± 0.9	0.626*
^Granulomas disappearance	7.2 ± 2.9	6.8 ± 2.8	0.760*

Characteristics of ostium granulomas			
Number	Ostia number (n, %)		*p* value
Single	0 (0)	22 (37.3)	** < 0.001**^**#**^
Multiple	12 (92.3)	21 (35.6)	
Diffuse	1 (7.7)	16 (27.1)	

Success rate	Ostia number (n, %)		*p* value
Anatomical	12 (92.3)	49 (83.1)	0.363^&^
Functional	12 (92.3)	53 (89.8)	0.629^&^

*Independent T-test. #Chi-square test. ^The total ostia number of granuloma disappearance was 69 (three ostia: one decreased and two stationary in size were excluded). ^&^Fisher’s exact test.

*p* values < 0.05 are considered significant and presented in bold type.

## Discussions

Ostium granuloma is well known as one of the most common factors that affect surgical outcomes after endoscopic DCR. Once ostium granulomas develop, the anatomical and functional success rates decrease significantly^10,13,19^. Many studies focused on the office-based surgical management of postoperative ostium granuloma, which reported satisfactory results^15,20^. However, the related cost and patient discomfort can never be overlooked. Repeated manipulation in the nasal cavity might also result in further damage to the normal nasal mucosa and lead to more unwanted cicatricial changes.

In the current study, the authors did not perform routine surgical removal for ostium granulomas that developed after endoscopic DCR surgeries. Through regular endoscopic follow-up, we disclosed the sequential changes of ostium granulomas and the final surgical success rate under the usage of topical intranasal steroid spray. We did not only point out the time point of granuloma development after the operation but also revealed the sequential regression course under intranasal steroid usage. Furthermore, we elucidated the role of a postoperative steroid regimen (topical nasal steroid spray with or without intralesional TA injection) in altering the sequential course of granuloma and the success rate of DCR surgery, although this study did not have a control group.

As one of the most common postoperative ostium-related complications, there was a wide range of reported ostium granuloma incidence (20–45.7%) due to a lack of strict and uniform standard surgical methods; moreover, this might also differ between experienced and non-experienced surgeons^6,21–25^. In addition, inserted silicone tubes may also result in contact granulomas owing to tissue reactions to foreign bodies^26^. Some studies adopted an antifibrotic agent, such as intraoperative mitomycin-C soaking or even postoperative mitomycin-C eyedrops to prevent granuloma formation^7,10,27,28^. We recruited patients who underwent surgeries by a single surgeon to enhance the consistency of surgical and postoperative evaluation methods.

Ali et al. described the possible mechanism and time sequences of granuloma formation by incorporating the concept of nasal mucosa wound healing process^17^. The granulation phase usually starts between 2–4 weeks after tissue damage, and macroscopic normalization takes place within 12–18 weeks. In our current study, the granulomas were mostly detected within 2 months postoperatively, while none of them were found after 6 months postoperatively. Our result also complied with the aforementioned healing mechanism observed by Ali et al.; thus, we speculated that conservative medical treatment with intranasal steroids could effectively modify the healing process of the nasal mucosa during an early postoperative period of 1–2 months. Steroids are potent in suppressing the recruitment of fibroblasts; therefore, they are widely used for reducing scar tissue formation postoperatively^29^. Therefore, all the patients were recommended to use nasal corticosteroid spray once daily for at least 1 month postoperatively.

In our study, the ostium granulomas were more commonly located superiorly (28 of 72, 38.9%), which was consistent with the findings in previous literature^11,16^. The phenomenon that ostium granulomas tend to develop superiorly to the ostium has its anatomical basis. For endoscopic DCR, it is important to ensure adequate exposure of the lacrimal sac and sufficient size of the osteotomy. In order to achieve an optimal result, the thicker and relatively more cephalic part of the frontal process of the maxilla has to be removed. Therefore, ideal mucosa-to-mucosa apposition is sometimes challenging to attain at the most superior part of the ostium, and the residual rough surface of a bare bone leads to subsequent granuloma occurrence^29^. Moreover, the frontal process of the maxilla of East Asians is not only thicker than that of Western ethnicity, but it is also significantly related to the surrounding bony structures adjacent to the lacrimal sac fossa^30^. The aforementioned anatomical features also predispose granulomas to reside at the superior quadrant of the ostium.

Some studies reported that the average granuloma detection time was 6 weeks postoperatively, and certain types of granulomas might affect the surgical outcomes; thus, excision or topical steroid treatment was suggested to optimize the DCR success rate^16,17^. However, in this study, the average interval between DCR operation and detection of granulomas was 2.4 ± 0.9 months (range: 1–6 months), which was later than that reported previously. Additionally, the characteristics of the granulomas, including location, shape, and number did not affect the final surgical success rate in our report. In our current cohort study, all the patients were asked to apply topical intranasal steroid spray once daily for at least 1 month. In contrast, other studies prescribed it only to specific patterns of ostium granulomas^16,17^. We speculated that it was the different principles of postoperative topical steroid regimens that led to such diversity. The adequate follow-up period should be at least > 6 months postoperatively in order to detect the potential development of ostium granulomas.

In our study, we found that a majority of the intranasally steroid-treated ostium granulomas resolved without surgical excision. The three ostia with residual refractory granulomas might be related to an insufficient follow-up period, while the residual granulomas did not affect the final surgical outcomes. The resolution of the unexcised ostium granulomas has been reported previously, but the precise time intervals from operation to granulomas resolution were not provided^16^. Even with the process and time interval for granuloma resolution were idiosyncratic and divergent between different individuals, optimal surgical prognosis could be anticipated with proper intranasal steroid regimen. Therefore, periodical endoscopic monitoring of ostium granuloma occurrence should be substantial in the aspect of steroid regimen adjustment, especially at 1–2 months after endoscopic DCR.

The consensus on treatment modalities for ostium granulomas after endoscopic DCR was still inconclusive. Some authors advocated that either medical or surgical treatment should be guided by the pattern of granulomas^16^, while some authors suggested that surgical excision under an office-based endoscopic view was favorable and less time-consuming with promising outcomes^15^. Some also proposed the early removal of silicone tubes in peritubal-type granulomas to prevent further proliferation of the granulation tissue^16^. Nevertheless, we routinely removed the tubes around 3 months postoperatively, and there should be a study for proper removal time of the silicone tubes after DCR in cases of granuloma development. Conservative treatment with postoperative mitomycin-C was also potent in the prevention of ostium granuloma formation^7,28^. However, the authors did not adopt mitomycin-C treatment, owing to side effects of mucosal irritation or possible delay in wound healing. In contrast to mitomycin-C, corticosteroids seemed to be a safe and effective choice for the prevention and treatment of ostium granulomas. Intralesional injections of steroids were preferred by some reports^11,16,29^, and we also adopted TA injections in patients with multiple granulomas, which caused impending blockade of the osteotomy. Nevertheless, the intralesional injections did not show significant benefits to the final surgical outcomes and granuloma disappearance rate in this report. Although the initial severity and extent of granulomas might be more significant for intralesional TA injection cases, we still could not draw a robust conclusion that intralesional TA injections were indeed advantageous. In contrast, the authors speculated that topical intranasal steroid spray regimen alone seemed not to be inferior to intralesional injections in the aspects of granuloma resolution and final surgical outcomes. Recently, medical practitioners should not only focus on the final treatment outcomes, but should consider patient satisfaction and medical costs. Moreover, repeated surgical manipulations could also lead to more severe nasal mucosal damage and cicatricial changes. Thus, the authors deferred routine surgical removal of ostium granulomas, considering the aforementioned concepts. Instead, we initiated a topical intranasal steroid regimen immediately after operation, and extended the regimen course if necessary. Intralesional steroid injections might aid in granuloma resolution, but whether they bring substantial benefits to outcome improvements is still obscure.

In conclusion, our current report is the first study to elucidate the sequential regression process and total resolution status of unexcised ostium granulomas under the coverage of topical intranasal steroid. Intranasal steroids usage was safe and well tolerated, and we did not observe any major adverse effect in our patients. We also speculated that minimal manipulation around the osteotomies could be more advantageous in the aspects of granuloma resolution and surgical prognosis. However, our current study has some limitations due to its retrospective and non-randomized comparative nature. Although the surgeries, silicone tubes removal, endoscopic follow-up, and regimen adjustment were all accomplished by a single surgeon, selection bias was sometimes inevitable. Additionally, the number of the intralesional TA injection group was relatively smaller than that of the non-injection group. More patient number enrollment is warranted to reach a more solid conclusion.

## Data Availability

The data that support the findings of this study are available on request from the corresponding authors (K.I.W).
